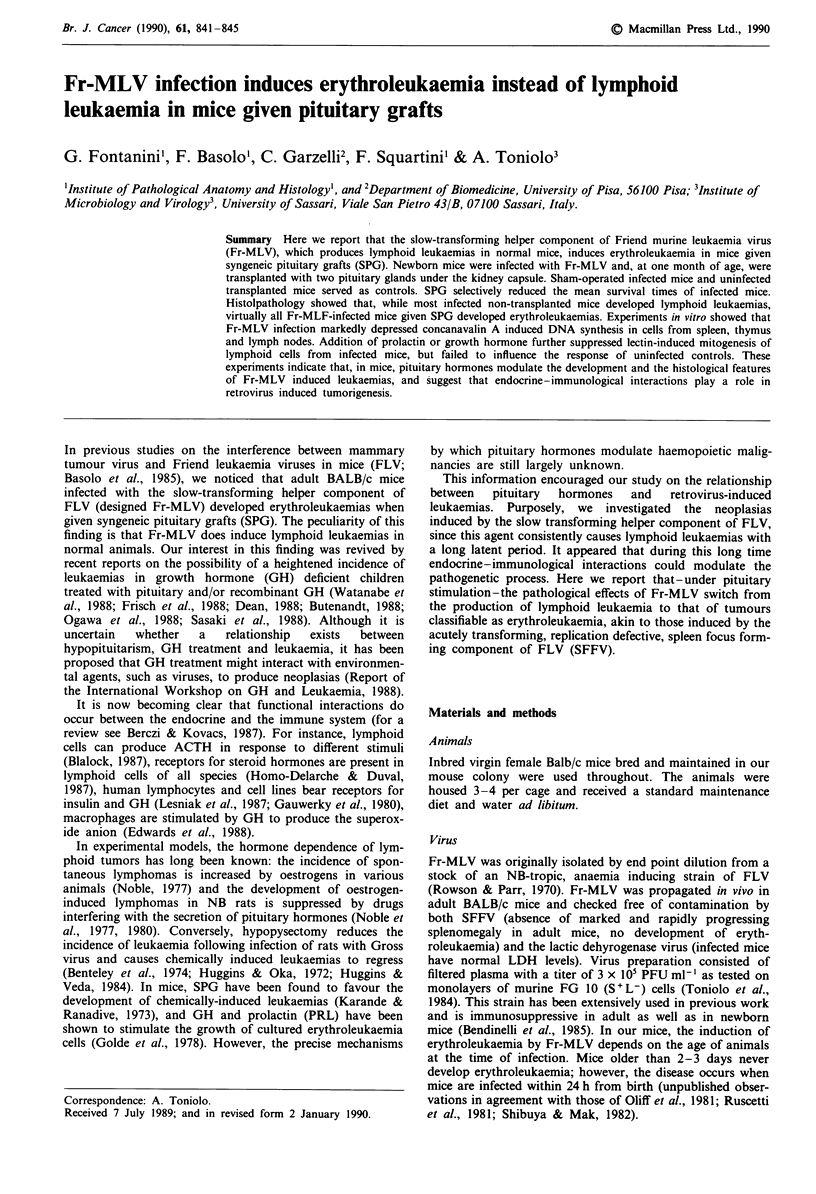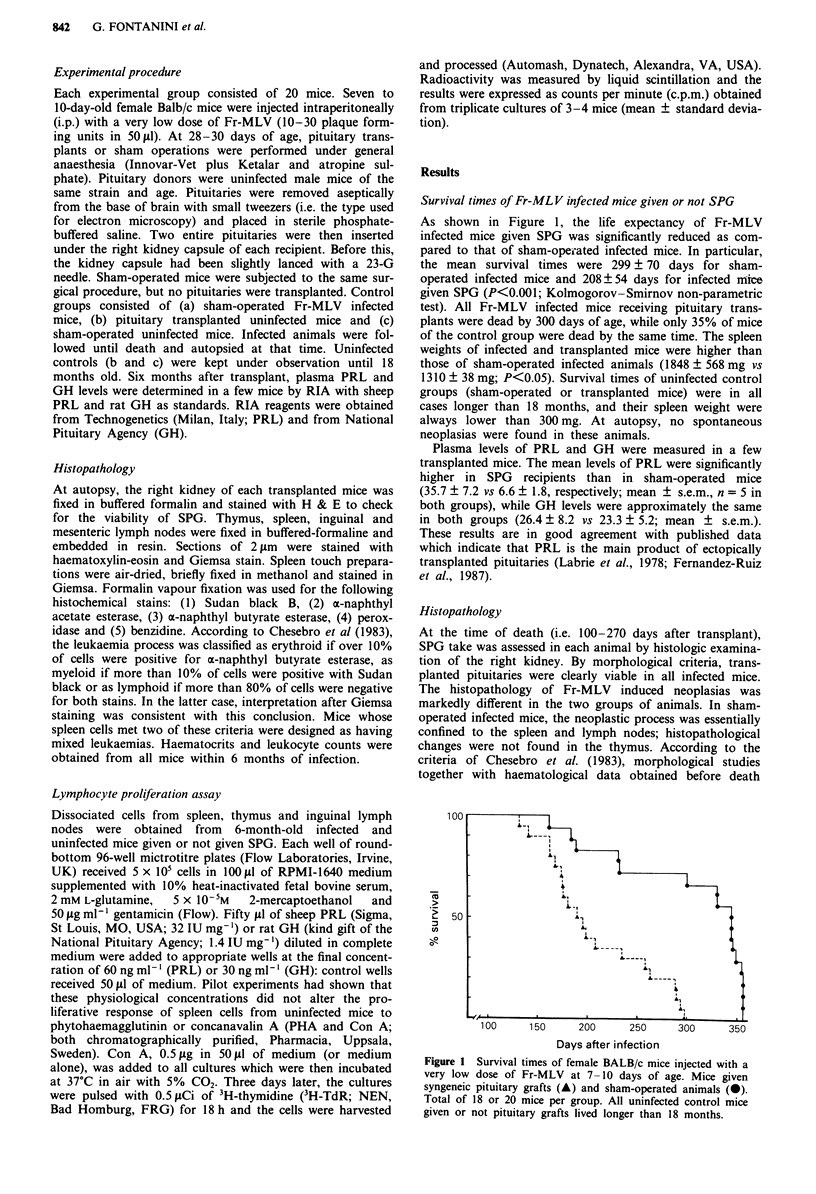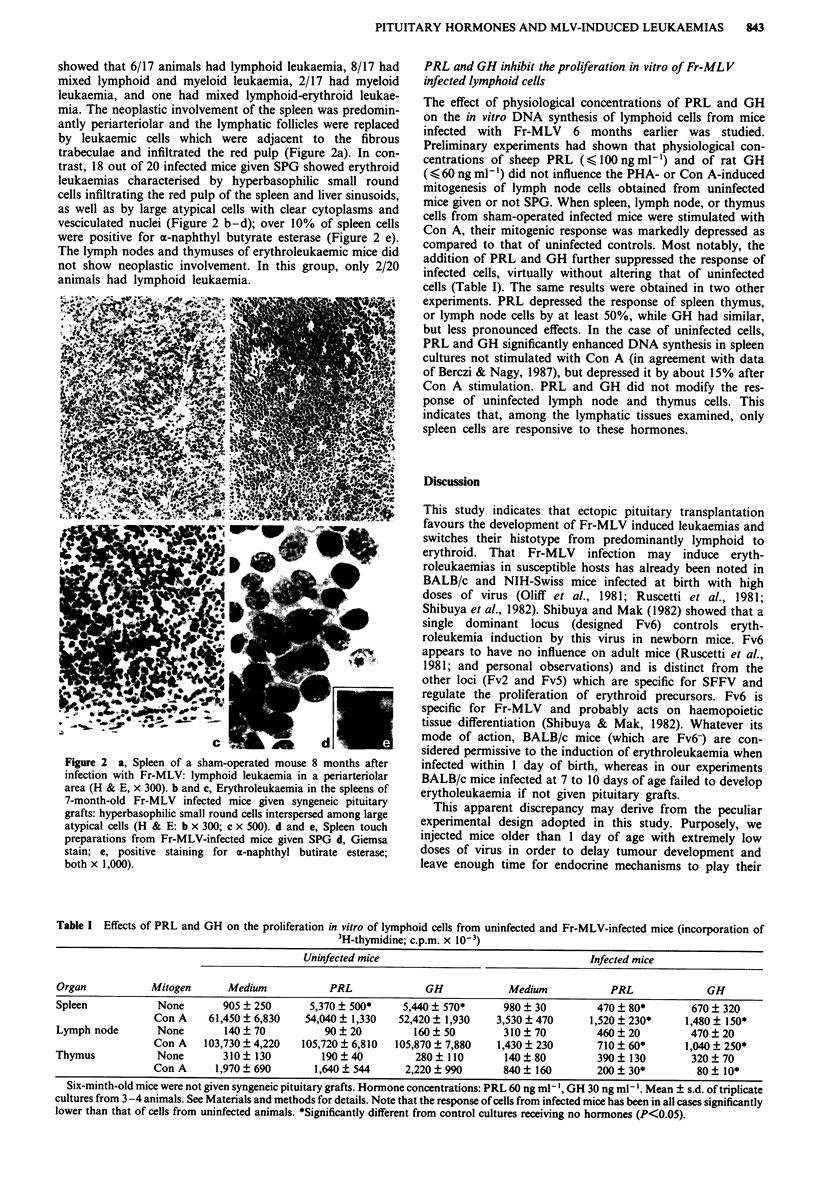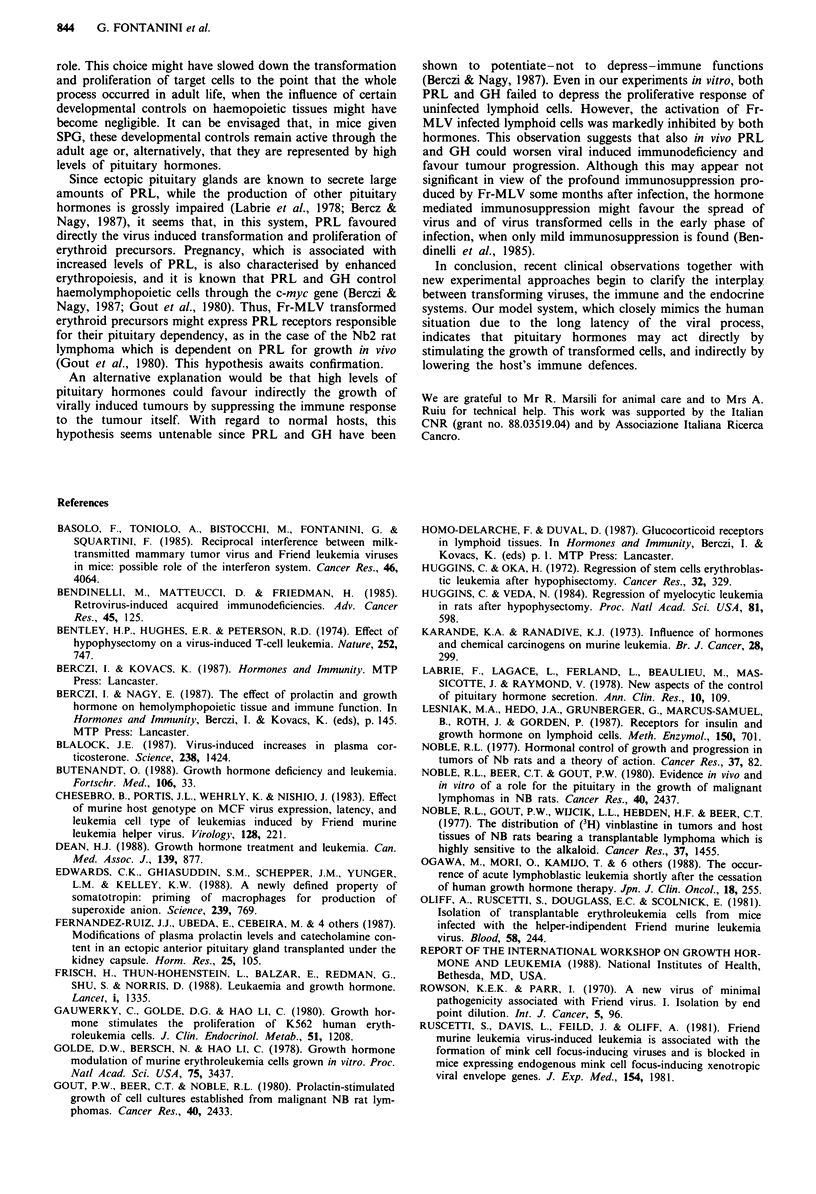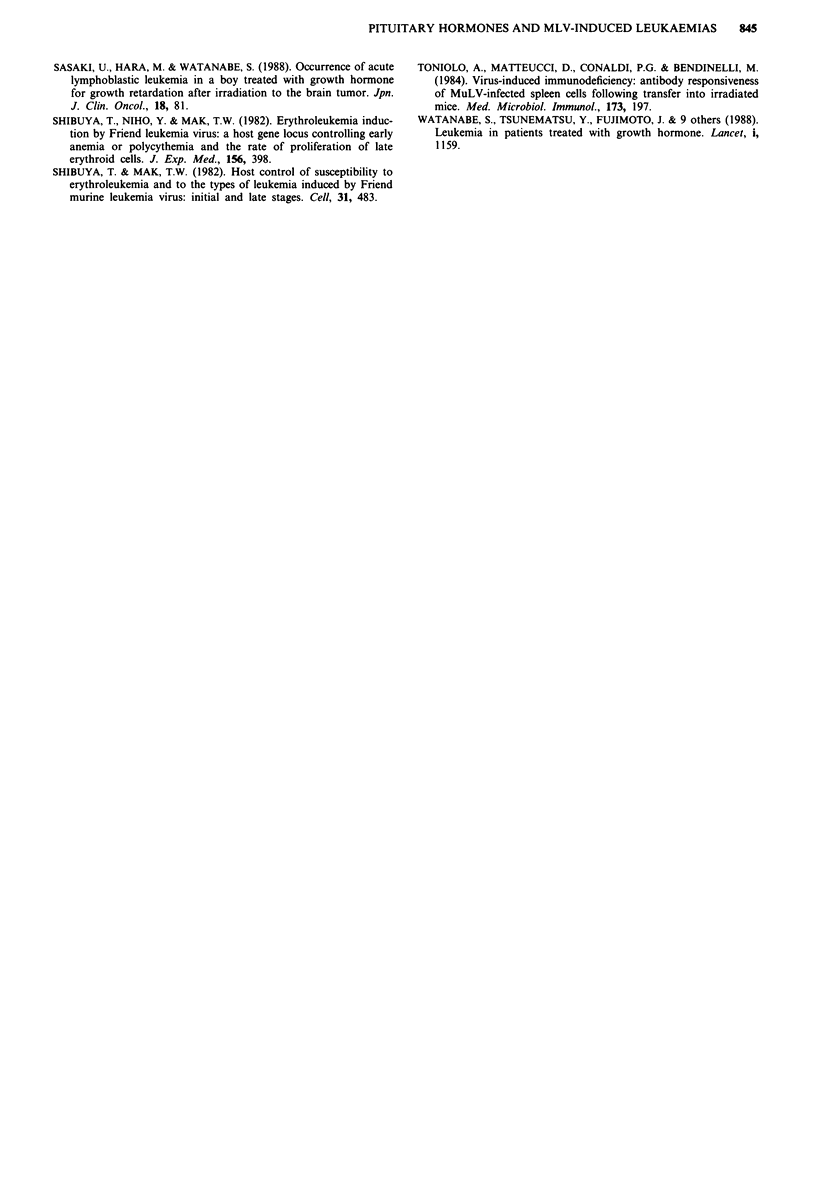# Fr-MLV infection induces erythroleukaemia instead of lymphoid leukaemia in mice given pituitary grafts.

**DOI:** 10.1038/bjc.1990.188

**Published:** 1990-06

**Authors:** G. Fontanini, F. Basolo, C. Garzelli, F. Squartini, A. Toniolo

**Affiliations:** Institute of Pathological Anatomy and Histology, University of Pisa, Italy.

## Abstract

**Images:**


*Br. J. Cancer* (1990), **61**, 841–845 © Macmillan Press Ltd., 1990

### Fr-MLV infection induces erythroleukaemia instead of lymphoid leukaemia in mice given pituitary grafts

G. Fontanini[1], F. Basolo[1], C. Garzelli[2], F. Squartini[1] & A. Toniolo[3]

[1]*Institute of Pathological Anatomy and Histology*[1], *and* [2]*Department of Biomedicine, University of Pisa, 56100 Pisa;* [3]*Institute of Microbiology and Virology*[3], *University of Sassari, Viale San Pietro 43/B, 07100 Sassari, Italy.*

**Summary** Here we report that the slow-transforming helper component of Friend murine leukaemia virus (Fr-MLV), which produces lymphoid leukaemias in normal mice, induces erythroleukaemia in mice given syngeneic pituitary grafts (SPG). Newborn mice were infected with Fr-MLV and, at one month of age, were transplanted with two pituitary glands under the kidney capsule. Sham-operated infected mice and uninfected transplanted mice served as controls. SPG selectively reduced the mean survival times of infected mice. Histolpathology showed that, while most infected non-transplanted mice developed lymphoid leukaemias, virtually all Fr-MLF-infected mice given SPG developed erythroleukaemias. Experiments *in vitro* showed that Fr-MLV infection markedly depressed concanavalin A induced DNA synthesis in cells from spleen, thymus and lymph nodes. Addition of prolactin or growth hormone further suppressed lectin-induced mitogenesis of lymphoid cells from infected mice, but failed to influence the response of uninfected controls. These experiments indicate that, in mice, pituitary hormones modulate the development and the histological features of Fr-MLV induced leukaemias, and suggest that endocrine–immunological interactions play a role in retrovirus induced tumorigenesis.

In previous studies on the interference between mammary tumour virus and Friend leukaemia viruses in mice (FLV; Basolo *et al.*, 1985), we noticed that adult BALB/c mice infected with the slow-transforming helper component of FLV (designed Fr-MLV) developed erythroleukaemias when given syngeneic pituitary grafts (SPG). The peculiarity of this finding is that Fr-MLV does induce lymphoid leukaemias in normal animals. Our interest in this finding was revived by recent reports on the possibility of a heightened incidence of leukaemias in growth hormone (GH) deficient children treated with pituitary and/or recombinant GH (Watanabe *et al.*, 1988; Frisch *et al.*, 1988; Dean, 1988; Butenandt, 1988; Ogawa *et al.*, 1988; Sasaki *et al.*, 1988). Although it is uncertain whether a relationship exists between hypopituitarism, GH treatment and leukaemia, it has been proposed that GH treatment might interact with environmental agents, such as viruses, to produce neoplasias (Report of the International Workshop on GH and Leukaemia, 1988).

It is now becoming clear that functional interactions do occur between the endocrine and the immune system (for a review see Berczi & Kovacs, 1987). For instance, lymphoid cells can produce ACTH in response to different stimuli (Blalock, 1987), receptors for steroid hormones are present in lymphoid cells of all species (Homo-Delarche & Duval, 1987), human lymphocytes and cell lines bear receptors for insulin and GH (Lesniak *et al.*, 1987; Gauwerky *et al.*, 1980), macrophages are stimulated by GH to produce the superoxide anion (Edwards *et al.*, 1988).

In experimental models, the hormone dependence of lymphoid tumors has long been known: the incidence of spontaneous lymphomas is increased by oestrogens in various animals (Noble, 1977) and the development of oestrogen-induced lymphomas in NB rats is suppressed by drugs interfering with the secretion of pituitary hormones (Noble *et al.*, 1977, 1980). Conversely, hypopysectomy reduces the incidence of leukaemia following infection of rats with Gross virus and causes chemically induced leukaemias to regress (Benteley *et al.*, 1974; Huggins & Oka, 1972; Huggins & Veda, 1984). In mice, SPG have been found to favour the development of chemically-induced leukaemias (Karande & Ranadive, 1973), and GH and prolactin (PRL) have been shown to stimulate the growth of cultured erythroleukaemia cells (Golde *et al.*, 1978). However, the precise mechanisms by which pituitary hormones modulate haemopoietic malignancies are still largely unknown.

This information encouraged our study on the relationship between pituitary hormones and retrovirus-induced leukaemias. Purposely, we investigated the neoplasias induced by the slow transforming helper component of FLV, since this agent consistently causes lymphoid leukaemias with a long latent period. It appeared that during this long time endocrine–immunological interactions could modulate the pathogenetic process. Here we report that–under pituitary stimulation–the pathological effects of Fr-MLV switch from the production of lymphoid leukaemia to that of tumours classifiable as erythroleukaemia, akin to those induced by the acutely transforming, replication defective, spleen focus forming component of FLV (SFFV).

### Materials and methods

### *Animals*

Inbred virgin female Balb/c mice bred and maintained in our mouse colony were used throughout. The animals were housed 3–4 per cage and received a standard maintenance diet and water *ad libitum.*

### *Virus*

Fr-MLV was originally isolated by end point dilution from a stock of an NB-tropic, anaemia inducing strain of FLV (Rowson & Parr, 1970). Fr-MLV was propagated *in vivo* in adult BALB/c mice and checked free of contamination by both SFFV (absence of marked and rapidly progressing splenomegaly in adult mice, no development of erythroleukaemia) and the lactic dehyrogenase virus (infected mice have normal LDH levels). Virus preparation consisted of filtered plasma with a titer of $3 \times 10^5$ PFU $ml^{-1}$ as tested on monolayers of murine FG 10 ($S^+L^-$) cells (Toniolo *et al.*, 1984). This strain has been extensively used in previous work and is immunosuppressive in adult as well as in newborn mice (Bendinelli *et al.*, 1985). In our mice, the induction of erythroleukaemia by Fr-MLV depends on the age of animals at the time of infection. Mice older than 2–3 days never develop erythroleukaemia; however, the disease occurs when mice are infected within 24 h from birth (unpublished observations in agreement with those of Oliff *et al.*, 1981; Ruscetti *et al.*, 1981; Shibuya & Mak, 1982).

Correspondence: A. Toniolo.
Received 7 July 1989; and in revised form 2 January 1990.

*Experimental procedure*

Each experimental group consisted of 20 mice. Seven to 10-day-old female Balb/c mice were injected intraperitoneally (i.p.) with a very low dose of Fr-MLV (10–30 plaque forming units in 50 µl). At 28–30 days of age, pituitary transplants or sham operations were performed under general anaesthesia (Innovar-Vet plus Ketalar and atropine sulphate). Pituitary donors were uninfected male mice of the same strain and age. Pituitaries were removed aseptically from the base of brain with small tweezers (i.e. the type used for electron microscopy) and placed in sterile phosphate-buffered saline. Two entire pituitaries were then inserted under the right kidney capsule of each recipient. Before this, the kidney capsule had been slightly lanced with a 23-G needle. Sham-operated mice were subjected to the same surgical procedure, but no pituitaries were transplanted. Control groups consisted of (a) sham-operated Fr-MLV infected mice, (b) pituitary transplanted uninfected mice and (c) sham-operated uninfected mice. Infected animals were followed until death and autopsied at that time. Uninfected controls (b and c) were kept under observation until 18 months old. Six months after transplant, plasma PRL and GH levels were determined in a few mice by RIA with sheep PRL and rat GH as standards. RIA reagents were obtained from Technogenetics (Milan, Italy; PRL) and from National Pituitary Agency (GH).

*Histopathology*

At autopsy, the right kidney of each transplanted mice was fixed in buffered formalin and stained with H & E to check for the viability of SPG. Thymus, spleen, inguinal and mesenteric lymph nodes were fixed in buffered-formaline and embedded in resin. Sections of 2 µm were stained with haematoxylin-eosin and Giemsa stain. Spleen touch preparations were air-dried, briefly fixed in methanol and stained in Giemsa. Formalin vapour fixation was used for the following histochemical stains: (1) Sudan black B, (2) α-naphthyl acetate esterase, (3) α-naphthyl butyrate esterase, (4) peroxidase and (5) benzidine. According to Chesebro *et al* (1983), the leukaemia process was classified as erythroid if over 10% of cells were positive for α-naphthyl butyrate esterase, as myeloid if more than 10% of cells were positive with Sudan black or as lymphoid if more than 80% of cells were negative for both stains. In the latter case, interpretation after Giemsa staining was consistent with this conclusion. Mice whose spleen cells met two of these criteria were designed as having mixed leukaemias. Haematocrits and leukocyte counts were obtained from all mice within 6 months of infection.

*Lymphocyte proliferation assay*

Dissociated cells from spleen, thymus and inguinal lymph nodes were obtained from 6-month-old infected and uninfected mice given or not given SPG. Each well of round-bottom 96-well mictrotitre plates (Flow Laboratories, Irvine, UK) received $5 \times 10^5$ cells in 100 µl of RPMI-1640 medium supplemented with 10% heat-inactivated fetal bovine serum, 2 mM L-glutamine, $5 \times 10^{-5}$M 2-mercaptoethanol and 50 µg ml$^{-1}$ gentamicin (Flow). Fifty µl of sheep PRL (Sigma, St Louis, MO, USA; 32 IU mg$^{-1}$) or rat GH (kind gift of the National Pituitary Agency; 1.4 IU mg$^{-1}$) diluted in complete medium were added to appropriate wells at the final concentration of 60 ng ml$^{-1}$ (PRL) or 30 ng ml$^{-1}$ (GH): control wells received 50 µl of medium. Pilot experiments had shown that these physiological concentrations did not alter the proliferative response of spleen cells from uninfected mice to phytohaemagglutinin or concanavalin A (PHA and Con A; both chromatographically purified, Pharmacia, Uppsala, Sweden). Con A, 0.5 µg in 50 µl of medium (or medium alone), was added to all cultures which were then incubated at 37°C in air with 5% $CO_2$. Three days later, the cultures were pulsed with 0.5 µCi of $^3$H-thymidine ($^3$H-TdR; NEN, Bad Homburg, FRG) for 18 h and the cells were harvested and processed (Automash, Dynatech, Alexandra, VA, USA). Radioactivity was measured by liquid scintillation and the results were expressed as counts per minute (c.p.m.) obtained from triplicate cultures of 3–4 mice (mean ± standard deviation).

### Results

*Survival times of Fr-MLV infected mice given or not SPG*

As shown in Figure 1, the life expectancy of Fr-MLV infected mice given SPG was significantly reduced as compared to that of sham-operated infected mice. In particular, the mean survival times were 299 ± 70 days for sham-operated infected mice and 208 ± 54 days for infected mice given SPG ($P$<0.001; Kolmogorov–Smirnov non-parametric test). All Fr-MLV infected mice receiving pituitary transplants were dead by 300 days of age, while only 35% of mice of the control group were dead by the same time. The spleen weights of infected and transplanted mice were higher than those of sham-operated infected animals (1848 ± 568 mg *vs* 1310 ± 38 mg; $P$<0.05). Survival times of uninfected control groups (sham-operated or transplanted mice) were in all cases longer than 18 months, and their spleen weight were always lower than 300 mg. At autopsy, no spontaneous neoplasias were found in these animals.

Plasma levels of PRL and GH were measured in a few transplanted mice. The mean levels of PRL were significantly higher in SPG recipients than in sham-operated mice (35.7 ± 7.2 *vs* 6.6 ± 1.8, respectively; mean ± s.e.m., $n$ = 5 in both groups), while GH levels were approximately the same in both groups (26.4 ± 8.2 *vs* 23.3 ± 5.2; mean ± s.e.m.). These results are in good agreement with published data which indicate that PRL is the main product of ectopically transplanted pituitaries (Labrie *et al*., 1978; Fernandez-Ruiz *et al*., 1987).

*Histopathology*

At the time of death (i.e. 100–270 days after transplant), SPG take was assessed in each animal by histologic examination of the right kidney. By morphological criteria, transplanted pituitaries were clearly viable in all infected mice. The histopathology of Fr-MLV induced neoplasias was markedly different in the two groups of animals. In sham-operated infected mice, the neoplastic process was essentially confined to the spleen and lymph nodes; histopathological changes were not found in the thymus. According to the criteria of Chesebro *et al*. (1983), morphological studies together with haematological data obtained before death

**Figure 1** Survival times of female BALB/c mice injected with a very low dose of Fr-MLV at 7–10 days of age. Mice given syngeneic pituitary grafts (▲) and sham-operated animals (●). Total of 18 or 20 mice per group. All uninfected control mice given or not pituitary grafts lived longer than 18 months.

showed that 6/17 animals had lymphoid leukaemia, 8/17 had mixed lymphoid and myeloid leukaemia, 2/17 had myeloid leukaemia, and one had mixed lymphoid-erythroid leukaemia. The neoplastic involvement of the spleen was predominantly periarteriolar and the lymphatic follicles were replaced by leukaemic cells which were adjacent to the fibrous trabeculae and infiltrated the red pulp (Figure 2a). In contrast, 18 out of 20 infected mice given SPG showed erythroid leukaemias characterised by hyperbasophilic small round cells infiltrating the red pulp of the spleen and liver sinusoids, as well as by large atypical cells with clear cytoplasms and vesciculated nuclei (Figure 2 b–d); over 10% of spleen cells were positive for α-naphthyl butyrate esterase (Figure 2 e). The lymph nodes and thymuses of erythroleukaemic mice did not show neoplastic involvement. In this group, only 2/20 animals had lymphoid leukaemia.

**Figure 2** **a**, Spleen of a sham-operated mouse 8 months after infection with Fr-MLV: lymphoid leukaemia in a periarteriolar area (H & E, × 300). **b** and **c**, Erythroleukaemia in the spleens of 7-month-old Fr-MLV infected mice given syngeneic pituitary grafts: hyperbasophilic small round cells interspersed among large atypical cells (H & E: **b** × 300; **c** × 500). **d** and **e**, Spleen touch preparations from Fr-MLV-infected mice given SPG **d**, Giemsa stain; **e**, positive staining for α-naphthyl butirate esterase; both × 1,000).

*PRL and GH inhibit the proliferation in vitro of Fr-MLV infected lymphoid cells*

The effect of physiological concentrations of PRL and GH on the *in vitro* DNA synthesis of lymphoid cells from mice infected with Fr-MLV 6 months earlier was studied. Preliminary experiments had shown that physiological concentrations of sheep PRL ($\leqslant 100\ ng\ ml^{-1}$) and of rat GH ($\leqslant 60\ ng\ ml^{-1}$) did not influence the PHA- or Con A-induced mitogenesis of lymph node cells obtained from uninfected mice given or not SPG. When spleen, lymph node, or thymus cells from sham-operated infected mice were stimulated with Con A, their mitogenic response was markedly depressed as compared to that of uninfected controls. Most notably, the addition of PRL and GH further suppressed the response of infected cells, virtually without altering that of uninfected cells (Table I). The same results were obtained in two other experiments. PRL depressed the response of spleen thymus, or lymph node cells by at least 50%, while GH had similar, but less pronounced effects. In the case of uninfected cells, PRL and GH significantly enhanced DNA synthesis in spleen cultures not stimulated with Con A (in agreement with data of Berczi & Nagy, 1987), but depressed it by about 15% after Con A stimulation. PRL and GH did not modify the response of uninfected lymph node and thymus cells. This indicates that, among the lymphatic tissues examined, only spleen cells are responsive to these hormones.

### Discussion

This study indicates that ectopic pituitary transplantation favours the development of Fr-MLV induced leukaemias and switches their histotype from predominantly lymphoid to erythroid. That Fr-MLV infection may induce erythroleukaemias in susceptible hosts has already been noted in BALB/c and NIH-Swiss mice infected at birth with high doses of virus (Oliff *et al.*, 1981; Ruscetti *et al.*, 1981; Shibuya *et al.*, 1982). Shibuya and Mak (1982) showed that a single dominant locus (designed Fv6) controls erythroleukemia induction by this virus in newborn mice. Fv6 appears to have no influence on adult mice (Ruscetti *et al.*, 1981; and personal observations) and is distinct from the other loci (Fv2 and Fv5) which are specific for SFFV and regulate the proliferation of erythroid precursors. Fv6 is specific for Fr-MLV and probably acts on haemopoietic tissue differentiation (Shibuya & Mak, 1982). Whatever its mode of action, BALB/c mice (which are $Fv6^{-}$) are considered permissive to the induction of erythroleukaemia when infected within 1 day of birth, whereas in our experiments BALB/c mice infected at 7 to 10 days of age failed to develop erytholeukaemia if not given pituitary grafts.

This apparent discrepancy may derive from the peculiar experimental design adopted in this study. Purposely, we injected mice older than 1 day of age with extremely low doses of virus in order to delay tumour development and leave enough time for endocrine mechanisms to play their

**Table I** Effects of PRL and GH on the proliferation *in vitro* of lymphoid cells from uninfected and Fr-MLV-infected mice (incorporation of $^{3}H$-thymidine; c.p.m. × $10^{-3}$)

| | | *Uninfected mice* | | | *Infected mice* | | |
|---|---|---|---|---|---|---|---|
| *Organ* | *Mitogen* | *Medium* | *PRL* | *GH* | *Medium* | *PRL* | *GH* |
| Spleen | None | 905 ± 250 | 5,370 ± 500* | 5,440 ± 570* | 980 ± 30 | 470 ± 80* | 670 ± 320 |
| | Con A | 61,450 ± 6,830 | 54,040 ± 1,330 | 52,420 ± 1,930 | 3,530 ± 470 | 1,520 ± 230* | 1,480 ± 150* |
| Lymph node | None | 140 ± 70 | 90 ± 20 | 160 ± 50 | 310 ± 70 | 460 ± 20 | 470 ± 20 |
| | Con A | 103,730 ± 4,220 | 105,720 ± 6,810 | 105,870 ± 7,880 | 1,430 ± 230 | 710 ± 60* | 1,040 ± 250* |
| Thymus | None | 310 ± 130 | 190 ± 40 | 280 ± 110 | 140 ± 80 | 390 ± 130 | 320 ± 70 |
| | Con A | 1,970 ± 690 | 1,640 ± 544 | 2,220 ± 990 | 840 ± 160 | 200 ± 30* | 80 ± 10* |

Six-minth-old mice were not given syngeneic pituitary grafts. Hormone concentrations: PRL 60 ng $ml^{-1}$, GH 30 ng $ml^{-1}$. Mean ± s.d. of triplicate cultures from 3–4 animals. See Materials and methods for details. Note that the response of cells from infected mice has been in all cases significantly lower than that of cells from uninfected animals. *Significantly different from control cultures receiving no hormones ($P < 0.05$).

role. This choice might have slowed down the transformation and proliferation of target cells to the point that the whole process occurred in adult life, when the influence of certain developmental controls on haemopoietic tissues might have become negligible. It can be envisaged that, in mice given SPG, these developmental controls remain active through the adult age or, alternatively, that they are represented by high levels of pituitary hormones.

Since ectopic pituitary glands are known to secrete large amounts of PRL, while the production of other pituitary hormones is grossly impaired (Labrie *et al.*, 1978; Bercz & Nagy, 1987), it seems that, in this system, PRL favoured directly the virus induced transformation and proliferation of erythroid precursors. Pregnancy, which is associated with increased levels of PRL, is also characterised by enhanced erythropoiesis, and it is known that PRL and GH control haemolymphopoietic cells through the c-*myc* gene (Berczi & Nagy, 1987; Gout *et al.*, 1980). Thus, Fr-MLV transformed erythroid precursors might express PRL receptors responsible for their pituitary dependency, as in the case of the Nb2 rat lymphoma which is dependent on PRL for growth *in vivo* (Gout *et al.*, 1980). This hypothesis awaits confirmation.

An alternative explanation would be that high levels of pituitary hormones could favour indirectly the growth of virally induced tumours by suppressing the immune response to the tumour itself. With regard to normal hosts, this hypothesis seems untenable since PRL and GH have been shown to potentiate–not to depress–immune functions (Berczi & Nagy, 1987). Even in our experiments *in vitro*, both PRL and GH failed to depress the proliferative response of uninfected lymphoid cells. However, the activation of Fr-MLV infected lymphoid cells was markedly inhibited by both hormones. This observation suggests that also *in vivo* PRL and GH could worsen viral induced immunodeficiency and favour tumour progression. Although this may appear not significant in view of the profound immunosuppression produced by Fr-MLV some months after infection, the hormone mediated immunosuppression might favour the spread of virus and of virus transformed cells in the early phase of infection, when only mild immunosuppression is found (Bendinelli *et al.*, 1985).

In conclusion, recent clinical observations together with new experimental approaches begin to clarify the interplay between transforming viruses, the immune and the endocrine systems. Our model system, which closely mimics the human situation due to the long latency of the viral process, indicates that pituitary hormones may act directly by stimulating the growth of transformed cells, and indirectly by lowering the host's immune defences.

We are grateful to Mr R. Marsili for animal care and to Mrs A. Ruiu for technical help. This work was supported by the Italian CNR (grant no. 88.03519.04) and by Associazione Italiana Ricerca Cancro.

### References

BASOLO, F., TONIOLO, A., BISTOCCHI, M., FONTANINI, G. & SQUARTINI, F. (1985). Reciprocal interference between milk-transmitted mammary tumor virus and Friend leukemia viruses in mice: possible role of the interferon system. *Cancer Res.*, **46**, 4064.

BENDINELLI, M., MATTEUCCI, D. & FRIEDMAN, H. (1985). Retrovirus-induced acquired immunodeficiencies. *Adv. Cancer Res.*, **45**, 125.

BENTLEY, H.P., HUGHES, E.R. & PETERSON, R.D. (1974). Effect of hypophysectomy on a virus-induced T-cell leukemia. *Nature*, **252**, 747.

BERCZI, I. & KOVACS, K. (1987). *Hormones and Immunity*. MTP Press: Lancaster.

BERCZI, I. & NAGY, E. (1987). The effect of prolactin and growth hormone on hemolymphopoietic tissue and immune function. In *Hormones and Immunity*, Berczi, I. & Kovacs, K. (eds), p. 145. MTP Press: Lancaster.

BLALOCK, J.E. (1987). Virus-induced increases in plasma corticosterone. *Science*, **238**, 1424.

BUTENANDT, O. (1988). Growth hormone deficiency and leukemia. *Fortschr. Med.*, **106**, 33.

CHESEBRO, B., PORTIS, J.L., WEHRLY, K. & NISHIO, J. (1983). Effect of murine host genotype on MCF virus expression, latency, and leukemia cell type of leukemias induced by Friend murine leukemia helper virus. *Virology*, **128**, 221.

DEAN, H.J. (1988). Growth hormone treatment and leukemia. *Can. Med. Assoc. J.*, **139**, 877.

EDWARDS, C.K., GHIASUDDIN, S.M., SCHEPPER, J.M., YUNGER, L.M. & KELLEY, K.W. (1988). A newly defined property of somatotropin: priming of macrophages for production of superoxide anion. *Science*, **239**, 769.

FERNANDEZ-RUIZ, J.J., UBEDA, E., CEBEIRA, M. & 4 others (1987). Modifications of plasma prolactin levels and catecholamine content in an ectopic anterior pituitary gland transplanted under the kidney capsule. *Horm. Res.*, **25**, 105.

FRISCH, H., THUN-HOHENSTEIN, L., BALZAR, E., REDMAN, G., SHU, S. & NORRIS, D. (1988). Leukaemia and growth hormone. *Lancet*, **i**, 1335.

GAUWERKY, C., GOLDE, D.G. & HAO LI, C. (1980). Growth hormone stimulates the proliferation of K562 human erythroleukemia cells. *J. Clin. Endocrinol. Metab.*, **51**, 1208.

GOLDE, D.W., BERSCH, N. & HAO LI, C. (1978). Growth hormone modulation of murine erythroleukemia cells grown *in vitro*. *Proc. Natl Acad. Sci. USA*, **75**, 3437.

GOUT, P.W., BEER, C.T. & NOBLE, R.L. (1980). Prolactin-stimulated growth of cell cultures established from malignant NB rat lymphomas. *Cancer Res.*, **40**, 2433.

HOMO-DELARCHE, F. & DUVAL, D. (1987). Glucocorticoid receptors in lymphoid tissues. In *Hormones and Immunity*, Berczi, I. & Kovacs, K. (eds) p. 1. MTP Press: Lancaster.

HUGGINS, C. & OKA, H. (1972). Regression of stem cells erythroblastic leukemia after hypophisectomy. *Cancer Res.*, **32**, 329.

HUGGINS, C. & VEDA, N. (1984). Regression of myelocytic leukemia in rats after hypophysectomy. *Proc. Natl Acad. Sci. USA*, **81**, 598.

KARANDE, K.A. & RANADIVE, K.J. (1973). Influence of hormones and chemical carcinogens on murine leukemia. *Br. J. Cancer*, **28**, 299.

LABRIE, F., LAGACE, L., FERLAND, L., BEAULIEU, M., MASSICOTTE, J. & RAYMOND, V. (1978). New aspects of the control of pituitary hormone secretion. *Ann. Clin. Res.*, **10**, 109.

LESNIAK, M.A., HEDO, J.A., GRUNBERGER, G., MARCUS-SAMUEL, B., ROTH, J. & GORDEN, P. (1987). Receptors for insulin and growth hormone on lymphoid cells. *Meth. Enzymol.*, **150**, 701.

NOBLE, R.L. (1977). Hormonal control of growth and progression in tumors of Nb rats and a theory of action. *Cancer Res.*, **37**, 82.

NOBLE, R.L., BEER, C.T. & GOUT, P.W. (1980). Evidence *in vivo* and *in vitro* of a role for the pituitary in the growth of malignant lymphomas in NB rats. *Cancer Res.*, **40**, 2437.

NOBLE, R.L., GOUT, P.W., WIJCIK, L.L., HEBDEN, H.F. & BEER, C.T. (1977). The distribution of ($^3$H) vinblastine in tumors and host tissues of NB rats bearing a transplantable lymphoma which is highly sensitive to the alkaloid. *Cancer Res.*, **37**, 1455.

OGAWA, M., MORI, O., KAMIJO, T. & 6 others (1988). The occurrence of acute lymphoblastic leukemia shortly after the cessation of human growth hormone therapy. *Jpn. J. Clin. Oncol.*, **18**, 255.

OLIFF, A., RUSCETTI, S., DOUGLASS, E.C. & SCOLNICK, E. (1981). Isolation of transplantable erythroleukemia cells from mice infected with the helper-indipendent Friend murine leukemia virus. *Blood*, **58**, 244.

REPORT OF THE INTERNATIONAL WORKSHOP ON GROWTH HORMONE AND LEUKEMIA (1988). National Institutes of Health, Bethesda, MD, USA.

ROWSON, K.E.K. & PARR, I. (1970). A new virus of minimal pathogenicity associated with Friend virus. I. Isolation by end point dilution. *Int. J. Cancer*, **5**, 96.

RUSCETTI, S., DAVIS, L., FEILD, J. & OLIFF, A. (1981). Friend murine leukemia virus-induced leukemia is associated with the formation of mink cell focus-inducing viruses and is blocked in mice expressing endogenous mink cell focus-inducing xenotropic viral envelope genes. *J. Exp. Med.*, **154**, 1981.

SASAKI, U., HARA, M. & WATANABE, S. (1988). Occurrence of acute lymphoblastic leukemia in a boy treated with growth hormone for growth retardation after irradiation to the brain tumor. *Jpn. J. Clin. Oncol.*, **18**, 81.

SHIBUYA, T., NIHO, Y. & MAK, T.W. (1982). Erythroleukemia induction by Friend leukemia virus: a host gene locus controlling early anemia or polycythemia and the rate of proliferation of late erythroid cells. *J. Exp. Med.*, **156**, 398.

SHIBUYA, T. & MAK, T.W. (1982). Host control of susceptibility to erythroleukemia and to the types of leukemia induced by Friend murine leukemia virus: initial and late stages. *Cell*, **31**, 483.

TONIOLO, A., MATTEUCCI, D., CONALDI, P.G. & BENDINELLI, M. (1984). Virus-induced immunodeficiency: antibody responsiveness of MuLV-infected spleen cells following transfer into irradiated mice. *Med. Microbiol. Immunol.*, **173**, 197.

WATANABE, S., TSUNEMATSU, Y., FUJIMOTO, J. & 9 others (1988). Leukemia in patients treated with growth hormone. *Lancet*, **i**, 1159.